# Supplementary figures and images for: The oncogene cyclin D1 promotes bipolar spindle integrity under compressive force
Source: PLoS One. 2024 Mar 13;19(3):e0296779. doi: 10.1371/journal.pone.0296779 (PMC10936824; doi:10.1371/journal.pone.0296779)

Raw Images for Figure 1A

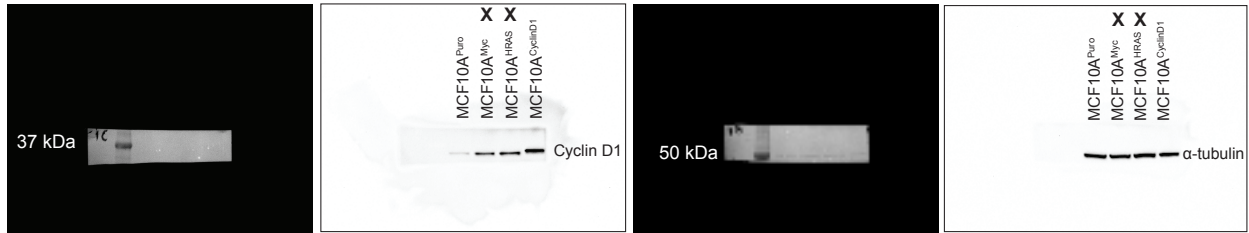

Raw Images for Figure 4A

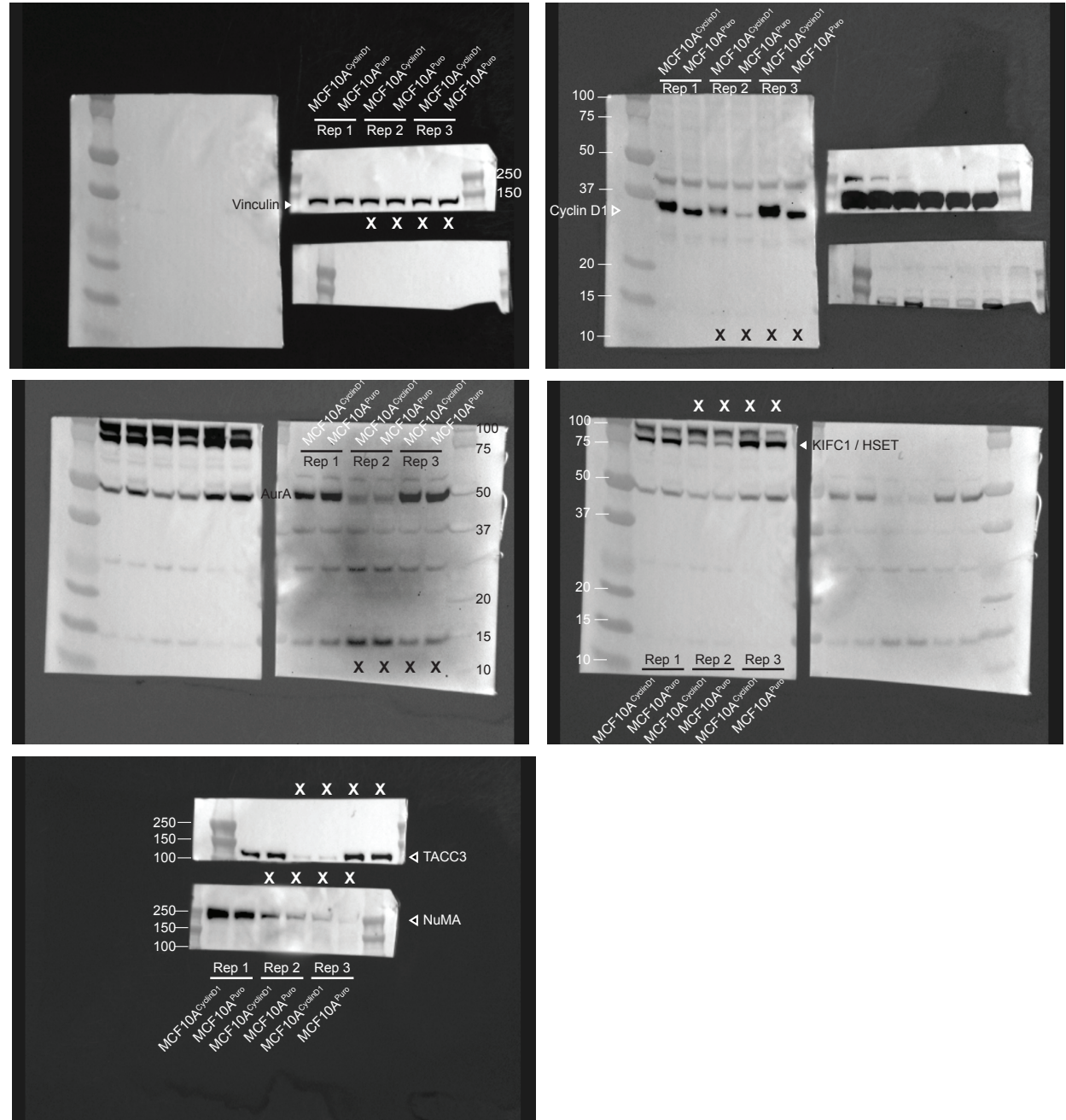

Supplement: S1 Raw images — (PDF) [file pone.0296779.s004.pdf]
